# Supplementary material for: Direct binding of phosphatidylglycerol at specific sites modulates desensitization of a ligand-gated ion channel
Source: eLife. 2019 Nov 14;8:e50766. doi: 10.7554/eLife.50766 (PMC6855808; doi:10.7554/eLife.50766)
Supplement: Supplementary file 1. — Table shows m/z, intensity, mass, and mass accuracy of each phospholipid species. [file elife-50766-supp1.docx]

**Supplementary File 1**

|  | **E coli** |  |  |  |  |
| --- | --- | --- | --- | --- | --- |
| **PE** | m/z | Intensity | Theo. Mass | Delta (mmu) | Composition |
| **14:0/14:0** | 634.4453 | 2.30E+04 | 634.4453 | -0.07 | C33 H65 O8 N P |
| **14:0/16:0** | 662.4765 | 1.70E+05 | 662.4766 | -0.11 | C35 H69 O8 N P |
| **16:0/16:1** | 688.4922 | 1.80E+05 | 688.4923 | -0.06 | C37 H71 O8 N P |
| **16:0/16:0** | 690.5079 | 2.20E+06 | 690.5079 | -0.06 | C37 H73 O8 N P |
| **16:0/17:1** | 702.5079 | 1.20E+06 | 702.5079 | -0.06 | C38 H73 O8 N P |
|  |  |  |  |  |  |
| **PG** | m/z | Intensity | Theo. Mass | Delta (mmu) | Composition |
| **16:0/16:1** | 719.4868 | 2.60E+05 | 719.4869 | -0.09 | C38 H72 O10 P |
| **16:0/16:0** | 721.5024 | 4.80E+05 | 721.5025 | -0.07 | C38 H74 O10 P |
| **16:0/17:1** | 733.5024 | 1.20E+06 | 733.5025 | -0.14 | C39 H74 O10 P |
| **16:1/18:1; 17:1/17:1** | 745.5023 | 3.10E+05 | 745.5025 | -0.17 | C40 H74 O10 P |
| **16:0/18:1** | 747.518 | 4.80E+05 | 747.5182 | -0.21 | C40 H76 O10 P |
| **17:1/18:1** | 759.5181 | 3.30E+05 | 759.5182 | -0.05 | C41 H76 O10 P |
| **17:1/18:0; 17:0/18:1** | 761.5337 | 1.30E+06 | 761.5338 | -0.1 | C41 H78 O10 P |
| **18:0/18:2** | 773.5337 | 6.30E+05 | 773.5338 | -0.1 | C42 H78 O10 P |
| **18:1/19:1** | 787.5494 | 2.80E+05 | 787.5495 | -0.1 | C43 H80 O10 P |
| **19:1/19:1** | 801.5649 | 2.60E+05 | 801.5651 | -0.18 | C44 H82 O10 P |
|  |  |  |  |  |  |
|  | **ELIC** |  |  |  |  |
| **PE** | m/z | Intensity | Theo. Mass | Delta (mmu) | Composition |
| **14:0/14:0** |  |  |  |  |  |
| **14:0/16:0** | 662.4769 | 9.30E+03 | 662.4766 | 0.3 | C35 H69 O8 N P |
| **16:0/16:1** | 688.4924 | 2.20E+04 | 688.4923 | 0.11 | C37 H71 O8 N P |
| **16:0/16:0** | 690.5078 | 8.30E+05 | 690.5079 | -0.12 | C37 H73 O8 N P |
| **16:0/17:1** | 702.5078 | 1.70E+05 | 702.5079 | -0.09 | C38 H73 O8 N P |
|  |  |  |  |  |  |
| **PG** | m/z | Intensity | Theo. Mass | Delta (mmu) | Composition |
| **16:0/16:1** | 719.4868 | 5.50E+04 | 719.4869 | -0.1 | C38 H72 O10 P |
| **16:0/16:0** | 721.5025 | 2.30E+05 | 721.5025 | -0.06 | C38 H74 O10 P |
| **16:0/17:1** | 733.5024 | 3.60E+05 | 733.5025 | -0.12 | C39 H74 O10 P |
| **16:1/18:1; 17:1/17:1** | 745.5024 | 1.80E+05 | 745.5025 | -0.14 | C40 H74 O10 P |
| **16:0/18:1** | 747.518 | 1.10E+05 | 747.5182 | -0.19 | C40 H76 O10 P |
| **17:1/18:1** | 759.5182 | 2.00E+06 | 759.5182 | 0.04 | C41 H76 O10 P |
| **17:1/18:0; 17:0/18:1** | 761.5338 | 4.00E+05 | 761.5338 | -0.04 | C41 H78 O10 P |
| **18:0/18:2** | 773.5338 | 3.20E+05 | 773.5338 | 0 | C42 H78 O10 P |
| **18:1/19:1** | 787.5494 | 1.80E+05 | 787.5495 | -0.04 | C43 H80 O10 P |
| **19:1/19:1** | 801.5651 | 9.10E+04 | 801.5651 | -0.03 | C44 H82 O10 P |

**Supplementary File 1:** Phophatidylethanolamine and phosphatidylglycerol species identified in lipid extracts by MS/MS. Table shows m/z, intensity, mass and mass accuracy of each phospholipid species.
